# Early outcomes of umbilical hernia repair with mesh suture

**DOI:** 10.1007/s10029-026-03798-1

**Published:** 2026-07-29

**Authors:** Taaha Hassan, Megan M Perez, Paige N Hackenberger, Gregory A Dumanian, Michael Shapiro

**Affiliations:** Department of Surgery, Northwestern Feinberg School of Medicine, 676 N. St. Clair Street, Suite 2320, Chicago, IL 60611 USA

**Keywords:** Umbilical hernia, Mesh suture, Duramesh, Abdominal wall closure

## Abstract

**Purpose:**

Mesh suture is a suture device designed to distribute pressure at the suture-tissue interface to decrease suture pull-through. The primary aim of this study was to evaluate the feasibility and short-term clinical outcomes in isolated umbilical hernia repairs.

**Methods:**

A retrospective cohort of patients undergoing umbilical hernia repair using mesh suture was analyzed between January 2023–2025. Patients were identified through institutional implant logs. Outcomes evaluated included surgical site infection (SSI), surgical site events (SSE), hernia recurrence, readmissions, and reoperations.

**Results:**

Fifty-eight isolated umbilical hernia repairs with mesh suture were performed by twenty-four surgeons. The mean hernia width was 1.7 cm (± 0.7). The 90-day SSI rate was 5.2% (*n* = 3), consisting of two superficial infections and one organ space infection. The 90-day SSE rate was 5.2% (*n* = 1 fascial dehiscence, *n* = 2 hematomas). There were seven readmissions (12.1%) within 90 days, of which only one was related to the abdominal wall closure. There were three reoperations within 90 days (5.2%): two hematoma washouts and one fascial dehiscence repair. The mean length of follow up was 594 days (± 284). There were no cases of hernia recurrence.

**Conclusions:**

Mesh suture provides the simplicity of suture repair with increased force distribution properties of mesh. In this initial multicenter experience, mesh suture demonstrated feasibility and acceptable short-term clinical outcomes for isolated umbilical hernia repair. Future randomized controlled trials comparing mesh suture versus planar mesh and standard suture are needed to compare techniques most effectively.

## Introduction

Umbilical hernias are the most common type of ventral hernia, with up to 25% of the general population possessing an asymptomatic hernia [1–3]. When symptomatic, umbilical hernias can have significant consequences including bowel obstruction, functional impairment, and pain. Additionally, many patients seek treatment because of the associated abdominal bulge and aesthetic concerns. In the United States alone, surgical repair of umbilical hernias accounts for nearly 175,000 procedures annually [4, 5].

The optimal repair for umbilical hernias remains debated [2]. A variety of surgical techniques have been described in the literature including open, laparoscopic, and robotic repairs. However, the most popular techniques involve the use of simple absorbable or permanent sutures, with the additional reinforcement with various planar mesh materials [6]. The recently published 2020 European Hernia Society guidelines recommend the use of planar mesh for open umbilical hernia repairs over 1 cm in diameter, citing five large meta-analyses that unanimously concluded that mesh decreases the recurrence rate without increasing surgical-site infections or postoperative pain [2]. For defects smaller than 1 cm, the evidence is limited, and a sutured repair may be considered in shared decision-making with the patient. However, open mesh repairs present additional technical challenges in comparison to standard suture including the need to open the defect wider for positioning of preperitoneal mesh and wider dissection of tissue planes for mesh overlays [7]. Thus, simple suture repair continues to be preferred by many surgeons due to its ease of use; in Germany, sutures alone are used in up to 75% of umbilical hernia repairs measuring less than 2 cm in diameter [8, 9]. The hypothesis of this study is the use of mesh suture in open umbilical hernia repair will have comparable complication rates to established outcomes for both sutures or planar mesh repairs as reported in the literature.

## Methods

### Data collection

An IRB approved retrospective cohort study was conducted between January 2023 and January 2025. Patients were identified through institutional implant logs of mesh suture, and all were treated at a single integrated hospital system anchored by a university hospital. Notably, the decision to utilize mesh suture was made by the operating surgeon as part of routine clinical practice, and no institutional protocol dictated the use of mesh suture versus alternative repair techniques during the study period. Inclusion criteria included the use of mesh suture for isolated open umbilical hernia repair. An isolated umbilical hernia was defined as a primary fascial defect located at the umbilical ring and not associated with additional abdominal wall hernias or concomitant abdominal wall reconstruction. Exclusion criteria included the additional use of planar mesh or concurrent abdominal procedure (such as abdominoplasty, prostatectomy, hysterectomy, kidney transplant). Retrospective review of the electronic medical record (EMR) was performed to evaluate patient characteristics, surgical details, and outcomes, including both the internal hospital system as well as a review of an all-external hospitals that can link to the EMR. The primary outcome for this study was incidence of surgical site infection (SSI) that included superficial, deep, and organ space infections. Surgical site events (SSE) were also collected including seroma, hematoma, soft tissue breakdown, cellulitis, suture granuloma, chronic draining sinus, and enterocutaneous fistula per definitions by Majumder et al. [10]. SSI, SSE, readmissions, and reoperations were recorded within one year of index surgery, however in cases where follow-up extended beyond 365 days, additional review of the medical record was performed to assess for chronic draining sinuses. Hernia formation after index surgery was recorded for longest follow up time available. Mean follow up was calculated from the index procedure to date of last documented review of the EMR. Hernia recurrence was defined as documentation of a recurrent defect based on physical exam or cross-sectional imaging. The EMR was reviewed for documented follow-ups, including both the internal hospital system as well as a review of the documentation of any external hospital that was available via the EMR. As mesh suture was used as part of standard clinical practice by the surgeon, patients did not give additional informed consent. Statistical analysis was performed using IBM SPSS Statistics 29 (IBM Corp., Armonk, NY). Descriptive statistics (median, mean, standard deviation) were performed to summarize patient characteristics, surgical details, and outcomes.

### Surgical Technique

**Fig. 1:**
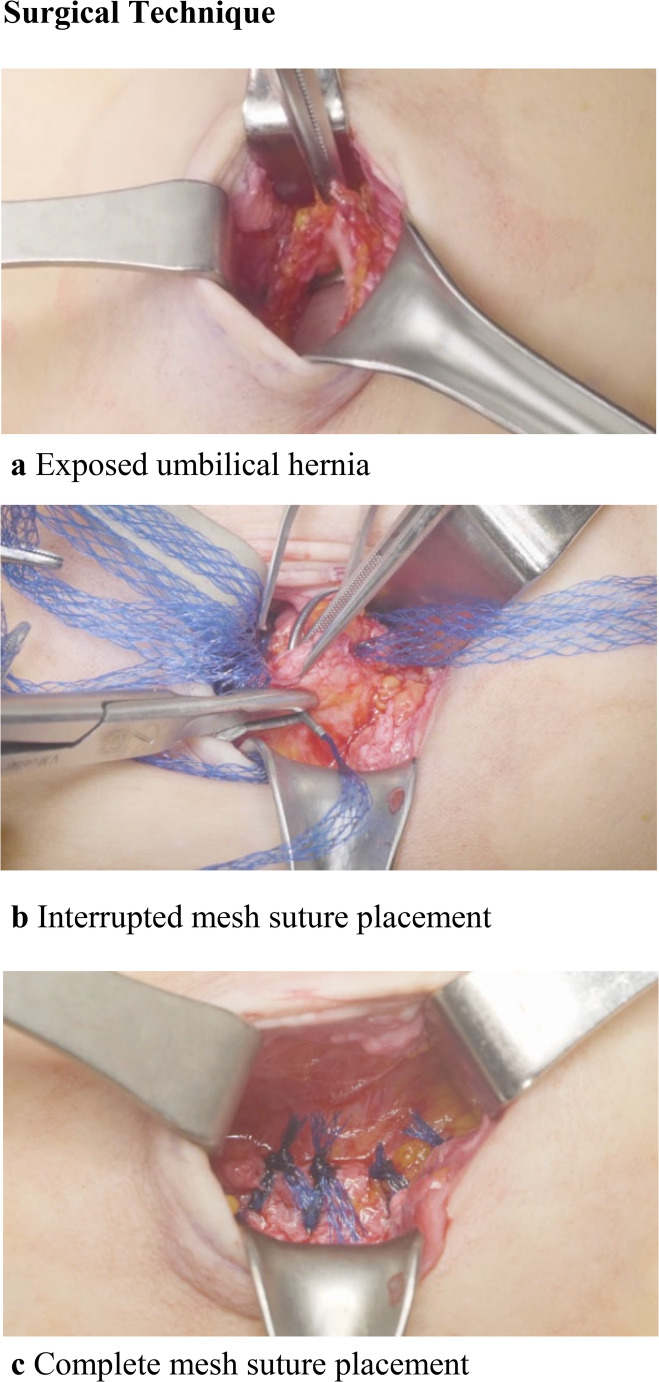
Complete mesh suture placement

Although mesh suture closure was performed by multiple surgeons, a broadly consistent technique is utilized across cases. The patient receives intravenous sedation, and approximately 10–15 cc of 1% xylocaine with epinephrine is used for local anesthesia. A curved incision is made at the curved inferior aspect of the umbilical stalk, and dissection is carried through the skin and subcutaneous tissues. The abdominal wall is cleared of soft tissue for a distance of 1 cm from 10 to 2 o’clock position in a circular dissection around the stalk (Fig. 1a). The base of the stalk is transected after confirming that just preperitoneal fat is contained within. Hernia contents are dissected free from the sac and reintroduced into the abdomen. The superior aspect of the stalk from 10 to 2 o’clock is then transected to clear the upper aspect of the abdominal wall defect of attached fat. The fascial defect is closed transversely with interrupted mesh sutures; all sutures were placed individually and tied collectively at the end with.

five alternating throws for each knot (Fig. 1b). Because mesh suture is composed of a macroporous mesh structure rather than a conventional monofilament filament, knot formation requires additional attention to knot seating. Following placement of each throw, the knot is progressively tightened and compressed (‘crimped’) to collapse the mesh structure and maximize friction between suture strands before placement of the subsequent throw. This seating process differs from conventional monofilament sutures and is important for achieving optimal knot security. The bite size is approximately 8 mm, and there are 5–6 mm between bites (Fig. 1c). Larger hernias were closed using running suture technique at the discretion of the surgeon. After tacking the base of the stalk back down to the abdominal wall, the skin closure is completed in two layers using 4 − 0 absorbable deep dermal sutures and a running 5 − 0 monofilament absorbable subcuticular suture. A drain may be used.

## Results

Fifty-eight isolated umbilical hernia repairs with mesh suture were performed by 24 surgeons across six surgical specialties (Table 1). Patients had a mean age of 52.2 (± 14.4) years and 58.6% of patients were male. The mean BMI was 29.7 (± 6.6). Patients had medical problems including diabetes (13.8%) and cancer (5.2%). About 27.6% of patients were former smokers and 6.9% were active smokers at time of surgery. Most patients (89.7%) had Centers for Disease Control (CDC) wound class I surgical fields. The mean preoperative hernia width was 1.7 cm (± 0.7). About one quarter (24.5%) of patients had at least one prior abdominal surgery. Two patients had a prior abdominal hernia repair, one umbilical hernia and one ventral hernia. The mean length of follow up was 594 days (± 284). All patient demographics are reported in Table 2. Fifteen (25.9%) patients had incarcerated contents at time of surgery. Of the six CDC class II-IV cases (clean-contaminated, contaminated, or dirty), the source of contamination was an open wound in one case and bowel manipulation in the remaining five. A variety of mesh suture/needle items were used (Table 3). The mean operative time was 91 min. The median length of inpatient stay was 0 days (IQR 0-0.75).

**Table 1:** Repairs by Surgeon Specialty

	*N* = 58 (%)
Primary surgeon specialty	
Plastics	14 (24.1)
General Surgery	21 (36.2)
Urology	1 (1.7)
Trauma	12 (20.7)
Colorectal	1 (1.7)
Transplant	9 (15.5)

**Table 2:** Patient Demographics

	*N* = 58 (%)
Age (years), (mean, SD)	52.2 ± 14.4
Race	
White	46 (79.3)
Black	4 (6.9)
Other	2 (3.4)
Declined	6 (10.3)
Male Gender	34 (58.6)
BMI (kg/m^2^), (mean, SD)	29.7 ± 6.6
Smoking status	
Active	4 (6.9)
Former	16 (27.6)
Never	38 (65.5)
Cancer	3 (5.2)
Chronic Obstructive Pulmonary Disease	2 (3.4)
Hypertension	21 (36.2)
Diabetes Mellitus	8 (13.8)
ASA classification	
I	3 (5.2)
II	31 (53.4)
III	21 (36.2)
IV	3 (5.2)
CDC wound classification	
Clean (CDC1)	52 (89.7)
Clean contaminated (CDC 2)	3 (5.2)
Contaminated (CDC 3)	2 (3.4)
Dirty or infected (CDC 4)	1 (1.7)
History abdominal surgery	17 (29.3)
History hernia repair	2 (3.4)
History mesh use	1 (1.7)
Length of follow up (days)	594.4 ± 283.5

**Table 3:** Surgical Details

	*N* = 58 (%)
Location of surgery	
NMH	43 (74.1)
Other	15 (25.8)
Length of inpatient stay (days) (median, IQR)	0 (0-0.75)
Incarcerated contents	15 (25.9)
Preoperative hernia width (cm) (mean, SD)	1.7 ± 0.7
Preoperative hernia area (cm^2^) (mean, SD)	3.3 ± 2.3
Contaminated source	
wound/skin breakdown	1 (1.7)
bowel manipulation	5 (8.6)
Surgical technique	
Interrupted	37 (63.8)
Running	15 (25.9)
Incision type	
Umbilical	51 (87.9)
Midline	7 (12.1)
Use of drains	4 (6.9)
Skin closure	
Suture	55 (94.8)
Staples	3 (5.2)
Operative time (minutes) (mean, SD)	91.1 ± 36.9
Mesh suture diameter-needle item size/USP size	
1-Large / 4 metric	5 (8.6)
1-Small / 4 metric	31 (53.4)
2-Large / 5 metric	2 (3.4)
2-Small / 5 metric	7 (12.1)
0-Large / 3.5 metric	9 (15.5)
0-Small / 3.5 metric	4 (6.9)

SSI/SSE outcomes are summarized in Table 4. The overall 90-day SSI rate was 5.2% and the SSE rate was 5.2%. All infections and surgical site events occurred in CDC I patients. Two patients (3.4%) had superficial infections, and one patient (1.7%) developed an organ space infection requiring interventional radiology-guided aspiration. Two patients (3.4%) developed hematomas, both of which required operative washout. In one case, mesh suture repair was removed (and later replaced) to access an intra-abdominal hematoma. In the second case, the hematoma was superficial and mesh suture repair was left intact. There was one case of fascial dehiscence (1.7%), which presented on postoperative day two with symptoms of bowel obstruction. Intraoperative findings revealed that the mesh suture knots had unraveled from a running repair. The defect was repaired using planar mesh.

**Table 4:** Surgical outcomes following repair

	*N* = 58 (%)
Surgical Site Infection (SSI)	
SSI total	3 (5.2)
SSI 0–90 days	3 (5.2)
Superficial infection	2 (3.4)
Deep infection	0 (0)
Organ space infection	1 (1.7)
SSI procedural intervention	1 (1.7)
Surgical Site Event (SSE)	
SSE total	3 (5.2)
SSE 0–90 days	3 (5.2)
Seroma	0 (0)
Hematoma	2 (3.4)
Soft tissue breakdown	0 (0)
Fascial dehiscence	1 (1.7)
Cellulitis	0 (0)
Suture granuloma	0 (0)
Chronic draining sinus	0 (0)
Enterocutaneous fistula	0 (0)
SSE procedural intervention	3 (5.2)
Readmissions within 1 year	9 (15.5)
0–90 days	7 (12.1)
Reoperations within 1 year	4 (6.9)
0–90 days	3 (5.2)
Death	0 (0)
Hernia recurrence	0 (0)
Duramesh removal	4 (6.9)
related to index surgery	2 (3.4)

Seven patients (12.1%) were readmitted within 90 days, with only one readmission related to the abdominal wall closure (previously described organ space infection). There were three reoperations within 90 days (5.2%), including the two hematoma washouts and the fascial dehiscence repair. One additional reoperation occurred within one year but was unrelated to the index procedure (cecal bascule repair). No hernia recurrences were documented. Four patients underwent removal of the index mesh suture: one during hematoma washout, one during repair of fascial dehiscence, and two during unrelated abdominal wall procedures (liver transplant and cecal bascule repair).

## Discussion

The primary aim of this study was to describe the technique of mesh suture repair for isolated umbilical hernias and report early clinical outcomes following its use in routine surgical practice. In this multicenter, multi-specialty retrospective cohort, mesh suture repair was associated with low rates of surgical site infection, surgical site events, readmission, reoperation, and clinically detected hernia recurrence during the available follow-up period. These findings provide early real-world outcome data for a novel repair technique that combines the simplicity of suture repair with the force-distribution properties of mesh. While the present study was not designed to directly compare mesh suture with conventional suture or planar mesh repairs, the observed outcomes provide preliminary evidence supporting the feasibility and safety of this approach and establish a foundation for future prospective comparative investigations.

Historically, standard suture closure of umbilical hernias was preferred given it is technically straightforward, limits tissue dissection and minimizes the amount of implanted foreign material. However, the hernia recurrence rate of these simple suture repairs has been reported to be upward of 21% [11–13]. One mechanism for repair failure is suture pull-through, in which the focused pressure at the suture-tissue interface causes cutting of tissue [14]. Given the unacceptable rate of hernia recurrence for suture-alone repairs, the use of planar mesh has been extensively studied for this indication. There have been five meta-analyses published that analyze postoperative outcomes in umbilical/epigastric hernia repairs when using planar mesh versus standard suture [5, 15–18]. Across all these studies, planar mesh repair was found to result in lower mean recurrence rates compared to standard suture repair (2.5% vs. 11.6%), without increasing complications such as SSIs, a feared complication in the setting of increased foreign body material. This evidence led to the guidelines developed in 2020 by the European and America Hernia Society that suggests repair with planar mesh is recommended for symptomatic umbilical and epigastric hernias greater than 1 cm [2]. Despite this evidence, surgeons still gravitate toward a suture only repair in many instances, either due to a small size of the defect or surgeon/patient preferences.

Mesh suture offers straightforward placement in the same fashion as simple suture with the advantage of reduced suture pull-through due to its unique suture geometry and properties. Specifically, mesh suture flattens across the tissue upon placement, thus increasing the overall surface area of tissue contact and allowing for better distribution of the tensile forces at the suture-tissue interface [19]. By definition, pressure is the force divided by the implant contact area. As mesh suture is wider than a standard suture, the axial force along the suture is distributed and the pressure experienced by the tissues lowered. Furthermore, the incorporation of a mesh design fosters fibrovascular incorporation of the foreign material that acts as a scar scaffold to increase the strength of the repair over time [20, 21]. In essence, the use of mesh suture offers the opportunity to combine the simplicity of suture repair with the force-distribution advantages of mesh to optimize hernia repairs [22]. The single fascial dehiscence observed in this cohort was attributed to knot unraveling. Although mesh suture is used in a manner like conventional suture, its unique mesh architecture influences knot formation and emphasizes the importance of appropriate knot seating during closure as described in the surgical technique section of the methods.

Regarding foreign material, mesh suture is made from a permanent polypropylene filament; however, the total amount of foreign material is far less than would be for a planar mesh [23]. In particular, risks of chronic draining sinus or fistula formation have been referred in the literature as significant in the use of permanent materials in contaminated fields, with reported rates of chronic draining sinus of up to 4.6% in planar mesh augmented repairs and 3.5% in permanent standard suture repairs [24–26]. Accordingly, in theory, mesh suture may be at higher risk for chronic draining sinuses compared with standard permanent sutures considering its increased surface area. However, mesh suture outcome studies have shown low rates of chronic infections, perhaps as a result of early tissue incorporation and increased fibrovascular ingrowth [21–23, 27]. Furthermore, mesh suture does not require wide dissection for placement as planar mesh does, thus, tissue vascularity is preserved which may also contribute to decreased infection and wound complications. Mesh strips are the predecessors of mesh suture and provide additional context to the advantages of mesh suture in umbilical hernia repair. A long-term mesh strip outcome study for umbilical hernias documented a single recurrence in 33 patients with 36 months of follow-up thought to be due to recurrent ascites [7]. Mesh strips have been supplanted with the availability of mesh suture as a Food and Drug Administration (FDA) product approved for marketing.

A recent meta-analysis by Madsen et al. compared aggregate outcomes in planar mesh repair and standard suture repair [28]. The aggregate rate of SSIs in planar mesh repair was 9.2% versus 5.7% in standard suture repair, as compared to 5.2% in our study with mesh suture. Similarly, 4.4% of planar mesh repair and 3.2% of standard suture repair surgeries resulted in SSEs, which are both comparable to the rate of 5.2% in the present mesh suture study. Interpretation of recurrence rates warrants additional caution, as clinically detected recurrences may underestimate the true incidence of repair failure. For example, Armañanzas et al. demonstrated a 32% incidence of trocar-site hernias identified by ultrasound following suture closure, highlighting the potential for clinically silent defects to go undetected without routine imaging surveillance [29]. No clinically detected hernia recurrences were identified in the present cohort during the available follow-up period, though there was one early fascial dehiscence secondary to knot unraveling as described previously. These findings should be interpreted as early data rather than evidence of comparative efficacy.

There are several limitations to recognize in this study. As a retrospective, descriptive cohort study, no control group was included. Therefore, the findings should be interpreted as early outcome data for mesh suture repair rather than evidence of superiority compared with conventional suture or planar mesh techniques. Furthermore, only patients undergoing mesh suture repair were included, and thus, the cohort may not be representative of all patients undergoing umbilical hernia repair during the study period at this integrated medical center. For example, 15.5% of the hernias were performed by the transplant surgery service, thus, mesh suture use may have been skewed towards a more complex patient phenotype. An additional limitation is the potential underestimation of hernia recurrence. Recurrence was defined by documentation of a recurrent fascial defect on physical examination or imaging obtained during routine clinical care. Consequently, small or asymptomatic recurrences that did not prompt imaging or were not detected on examination may have been missed. Furthermore, recurrences that did not prompt medical re-evaluation cannot be accounted for. As a result, the reported recurrence rate likely reflects early clinically detected recurrences rather than the true radiographic incidence of recurrence. The mean follow up period was less than two years, with six patients (10.3%) having less than 90 days of documented follow-up. Thus, the present study should be interpreted as reporting early outcomes, and long-term outcomes including chronic infections and recurrence rates require further investigation. In addition, procedures were performed by 24 surgeons with varying levels of experience using mesh suture. Multiple mesh suture sizes were utilized, and both interrupted and running closure techniques were employed. Consequently, variation in surgeon familiarity with the device and individual technical preferences may have influenced outcomes. Future studies with more standardized operative protocols may better define the effect of technique-specific factors on clinical outcomes. Future randomized controlled trials comparing mesh suture versus planar mesh and standard suture are needed to compare techniques most effectively and are underway.

## Conclusions

The use of mesh suture in umbilical hernia repairs appears feasible with early surgical complications comparable to reported rates to standard suture and planar mesh repairs. Future studies examining the comparative outcomes of conventional repair techniques to mesh suture with patient randomization are needed to understand long-term outcomes and efficacy. 
